# Transcriptomic analysis and carbohydrate metabolism-related enzyme expression across different pH values in *Rhizopus delemar*

**DOI:** 10.3389/fmicb.2024.1359830

**Published:** 2024-03-06

**Authors:** Jinpeng Liang, Yulan Chen, Sisi Li, Dongyang Liu, Hong Tian, Quanju Xiang, Ke Zhao, Xiumei Yu, Qiang Chen, Hongzhu Fan, Lingzi Zhang, Petri Penttinen, Yunfu Gu

**Affiliations:** ^1^Department of Microbiology, College of Resources, Sichuan Agricultural University, Chengdu, China; ^2^Liangshan Tobacco Corporation of Sichuan Province, Xichang, China; ^3^Institute of Agricultural Resources and Environmental Science, Sichuan Academy of Agricultural Sciences, Chengdu, China

**Keywords:** environmental pH, *Rhizopus delemar*, CAZymes, transcriptomics, growth and metabolism

## Abstract

**Introduction:**

pH is one of the important factors affecting the growth and performance of microorganisms.

**Methods:**

We studied the pH response and plant growth-promoting (PGP) ability of *Rhizopus delemar* using cultivation experiments and transcriptomics, and verified the expression profiles using quantitative real-time PCR.

**Results:**

pH affected the growth and PGP properties of *R. delemar*. At pH 7, the growth rate of *R. delemar* was rapid, whereas pH 4 and 8 inhibited mycelial growth and PGP ability, respectively. In the pot experiment, the plant height was the highest at pH 7, 56 cm, and the lowest at pH 4 and pH 5, 46.6 cm and 47 cm, respectively. Enzyme activities were highest at pH 6 to pH 7. Enzyme activities were highest at pH 6 to pH 7. Among the 1,629 differentially expressed genes (DEGs), 1,033 genes were up-regulated and 596 were down-regulated. A total of 1,623 DEGs were annotated to carbohydrate-active enzyme coding genes.

**Discussion:**

The PGP characteristics, e.g., Phosphorus solubilization ability, of *R. delemar* were strongest at pH 7. The results provide useful information regarding the molecular mechanism of *R. delemar* pH response.

## 1 Introduction

The fungus *Rhizopus delemar* (*R. delemar*) in the phylum Zygomycota is widely ustilized in industrial production due to its broad metabolic capacity. *R. delemar* is employed in ethanol production from starchy substrates and in the synthesis of organic acids such as lactic and fumaric acid from starch, cellulose, and hemicellulose (Zhou et al., 2011; Odoni et al., 2017). In addition, *R. delemar* strains exhibit plant growth promoting (PGP) and plant disease-suppressing abilities, which hold promise for sustainable agricultural applications (Zhang et al., 2022).

The PGP abilities of fungi include secreting organic acids to dissolve insoluble minerals and facilitate plant nutrient absorption, including nitrogen and phosphorus (Whitelaw et al., 1999). Moreover, PGP fungi may promote plant growth via phytohormone production, stress alleviation and suppression of pathogenic microorganisms (Hashem et al., 2019). These PGP abilities are governed by the organism’s genetic makeup. Notably, three PGP *Aspergillus* strains closely related to *A. puulaauensis* and *A. sydowii* in Ascomycota were found to carry more carbohydrate-active enzyme (CAZymes) genes, small secreted protein (SSPs) genes, and gene clusters encoding indole metabolism compared to pathogenic *Aspergillus* strains (Jing et al., 2022). To our knowledge, little attention has been given to exploring the PGP properties and related genes in zygomycetes.

Microorganisms exhibit sensitivity to environmental pH due to direct contact with the surroundings (Selvig et al., 2011). Fungi demonstrate adaptability to a wide pH range, maintaining homeostasis under acidic and alkaline conditions. For example, *A. nidulans* displays growth across pH 2.5 to 9.0 (Caddick et al., 1986). Environmental pH influences enzyme activities, thereby affecting microbial metabolism. In addition, pH affects cell membranes, enzyme-substrate affinity, and substrate absorption by microorganisms (Li et al., 2019). Microorganisms regulate gene expression to coordinate metabolic reactions in response to environmental conditions (Wilson et al., 2017), with specific gene expression modulated by environmental pH (Manteau et al., 2003). Fungi colonize and invade fibrous plant materials, participating in cell wall polysaccharide degradation and fiber digestion (Hébraud and Fèvre, 1988). Changes in pH lead to changes in the activities of glucosidase, cellulase and other CAZymes (Lou et al., 2013) that play key roles in the synthesis or decomposition of complex carbohydrates by degrading, modifying, and generating glycosidic bonds (Cantarel et al., 2009; Davies and Williams, 2016). In addition, CAZymes may contribute to PGP ability (Jing et al., 2022). To the best of our knowledge, there are no studies on the effect of pH on *R. delemar* CAZymes.

CAZymes are categorized into six groups based on associated catalytic activity motifs and domains: glycosyl hydrolases (GHs), carbohydrate esterases (CEs), polysaccharide lyases (PLs), carbohydrate-binding modules (CBMs), glycosyl transferases (GTs), and auxiliary activities (AAs) (Várnai et al., 2014; Drula et al., 2022). In the *R. oryzae* genome, CAZymes gene composition differs from most ascomycetes and basidiomycetes, suggesting *R. oryzae*’s utilization of easily digestible sugars but not complex plant cell wall polysaccharides, aligning with its growth profile (Battaglia et al., 2011). However, information regarding CAZymes in *R. delemar* and the effects of pH on the *R. delemar* transcriptome remains scarce.

We studied the plant growth-promoting (PGP) ability of *R. delemar* using transcriptomics to elucidate the PGP mechanism and the role of CAZymes therein, providing a theoretical foundation for further agricultural applications of *R. delemar*.

## 2 Materials and methods

### 2.1 Strain

The strain *Rhizopus delemar* SICAUZ-1 was isolated from corn (*Zea mays* L. var. Kangnongyu007) in Liangshan Yi Autonomous Region, China. The strain was maintained on potato dextrose agar (PDA). The partial ribosomal RNA gene sequence of *R. delemar* SICAUZ-1 is deposited in NCBI GenBank under the accession number ON584326. The strain was preserved in the China Center for Type Culture Collection under the identification number M2022700. The conidia of the isolates were stored at −80°C in 20% glycerol solution until use.

### 2.2 Determination of mycelial growth

To measure mycelial growth rate, mycelium was inoculated onto potato dextrose agar (PDA) and into 150 mL potato dextrose broth (PDB) medium (glucose 20 g/L, potato extract powder 300 g/L), followed by incubation at 28°C. Mycelial growth rate was determined on PDA by measuring the colony diameter every 12 h. Mycelial biomass was determined by growing the inoculant in PDB at 180 rpm for 72 h, after which the culture was filtered, and the weight of the mycelia was measured.

### 2.3 Plant growth-promoting characteristics of *Rhizopus delemar*

The inocula for the PGP ability tests were prepared by cultivating the strain in PDB medium at 28°C and 180 rpm until reaching the logarithmic phase. After the mycelium had colonized the medium completely within 3 days, the culture was centrifuged at 12,000 g for 1 min, and the pellet was re-suspended to a density of 7.50 × 10^7^ cfu/mL in ddH_2_O.

In the PGP ability tests, the pH of the media was adjusted to pH 4, 5, 6, 7, and 8 using 1 mol/L sodium hydroxide (NaOH) or 1 mol/L hydrochloric acid (HCl). All tests were conducted in triplicate. After sterilization, the pH value of the media was measured and adjusted when needed.

Indole-3-acetic acid (IAA) production was estimated as described by Joshi et al. (2021). 0.5 mL of the inoculant was inoculated into 150 mL PDB medium with 2 mg/ mL l-tryptophan and incubated at 150 rpm and 28°C for 72 h. After incubation, the culture was centrifuged at 10,000 *g* for 30 min, and 1 mL of the supernatant was mixed with 2 mL of Salkowski reagent (1 mL 0.5 M FeCl_3_ and 49 mL of 35% HClO_4_). After incubation at room temperature for 0.5 h, the concentration of IAA was determined using ultraviolet–visible spectrophotometry (Shimadzu, CHN) at 530 nm and a 1–50 μg/mL IAA standard curve.

To measure phosphorus solubilization capacity, 0.4 mL of the inoculant was inoculated into 150 mL inorganic phosphorus liquid medium (Phosphate solubilizer) and incubated at 180 rpm and 28°C for 72 h (Johri et al., 1999), followed by centrifugation of 1 mL of the culture at 10,000 rpm for 10 min, and measuring water-soluble phosphate concentrations in the supernatant using molybdenum antimony resistance colorimetry.

To determine siderophore production, 0.5 mL of the inoculant was inoculated on chrome azurol S (CAS) agar, and the plates were incubated at 28°C for 2 days (Machuca et al., 2003). An orange halo around a colony indicated the production of siderophores and the diameter of the halo indicated the amount produced (Schwyn and Neilands, 1987).

The *in vivo* PGP ability of the strain *Rhizopus delemar* SICAUZ-1 on corn (*Zea mays* L. var. Kangnongyu007) was tested in a greenhouse experiment. Particle size 1 mm quartz sand was sterilized at 121°C for 2 h. Corn seeds were washed with ddH_2_O and germinated in the dark for 2 days. The seeds were soaked for 2 h in 150 mL of the fungal suspension; soaking in sterile PDB served as the uninoculated control. Five seeds per pot were planted in plastic pots filled with 500 g of quartz sand. The pots were watered daily with Hoagland’s nutrient solution. After 45 days, the plants were carefully removed from the pots, the root and aboveground parts were separated and washed with distilled water, and dried until constant weight to determine the dry weight.

### 2.4 RNA extraction, cDNA library construction, and sequencing

The strain was cultivated as in the PGP tests at pH 4, 5, 6, 7, and 8, immediately frozen in liquid nitrogen and stored at −80°C. The mycelia from plates were pooled and ground to powder with a pestle in liquid nitrogen-chilled mortars. Cells were crushed using a FastPrep-24 (MP Biomedical, Shanghai, China). RNA was extracted using the Trizol Reagent (Invitrogen Life Technologies, Carlsbad, CA, United States). The concentration and quality of the extracted RNA were determined using a NanoDrop spectrophotometer (Thermo Scientific, Waltham, MA, United States). The integrity of the extracted RNA was assessed with gel electrophoresis in 1% agarose.

The TruSeq RNA Sample Preparation Kit (Illumina, San Diego, CA, United States) was used to create sequencing libraries according to the manufacturer’s instructions. Briefly, mRNA in 3 μg of RNA was purified using poly-T-oligo-attached magnetic beads, and fragmented in the fragmentation buffer utilizing divalent cations at a high temperature. First-strand cDNA was synthesized using SuperScript II and random oligonucleotides, and second-strand cDNA was synthesized using RNase H and DNA polymerase I. Exonuclease/polymerase activities were used to blunt the residual overhangs, and the enzymes were eliminated. Illumina paired-end adaptor oligonucleotides were ligated to prepare for hybridization after the 3′ ends of the DNA fragments were acetylated. The library fragments were purified using an AMPure XP system (Beckman Coulter, Beverly, CA, United States) to choose cDNA fragments of the preferred 200 bp length. In a 15-cycle PCR reaction, DNA fragments with ligated adaptors on both ends were specifically enriched using Illumina PCR Primer Cocktail. Products were measured using the Agilent high-sensitivity DNA test on a Bioanalyzer 2,100 system (Agilent) and purified using the AMPure XP system. The sequencing was carried out at Shanghai Personal Biotechnology Co. Ltd. (Shanghai, China) using an Illumina Hiseq platform.

### 2.5 Transcriptome data analysis

Sequences were trimmed and filtered to remove reads with average quality below Q20. The filtered reads were aligned to the *R. delemar* reference genome (GCA_011763715.1_ASM1176371v1_genomic.fna) using HISAT2 (Kim et al., 2015).

Transcripts were functionally annotated against the UniProt Consortium (2023), the Gene Ontology (GO) (2023), and the Kyoto Encyclopedia of Genes and Genomes (KEGG) (Kanehisa et al., 2000) databases using BLAST (Altschul et al., 1990). The annotated genes were assigned to GO categories. Unigenes were subjected to KEGG Orthology analysis using the KOBAS 2.0 web server.^1^

Based on the results on the PGP properties, differential expression analysis was conducted using DESeq2 (Wang et al., 2019) by comparing the expression at pH 7 to expression at other pH treatments. *p*-values in individual comparisons were adjusted for multiple testing using the procedure described by Wright (1992). Genes with |log2FoldChange| > 1 and *p* < 0.05/4 (the number of comparisons) were taken as differentially abundant. KEGG pathway enrichment analysis and GO functional enrichment analysis of differentially expressed genes (DEGs) were carried out using the KEGG biological pathway database^2^ and Gene Ontology^3^ databases, respectively.

### 2.6 Enzyme assay

The cultured fungal solution was centrifuged at 10,000 rpm for 10 min, and the precipitate was washed with 0.9% NaCl solution cells 3 times, suspended in phosphate buffer (100 mM, pH 7.4), treated with ultrasound for 5 min, centrifuged at 10,000 rpm and 4°C for 15 min, and the supernatant was collected as the crude enzyme extract. Lytic polysaccharide monooxygenases, protease, endo- and exo-1,4-beta-glucanase, β-glucosidase, and pectin lyase activities were determined as previously described (Yeo et al., 2007; Songulashvili et al., 2008; MacDonald et al., 2016; Rueda-Mejia et al., 2021).

### 2.7 CAZymes annotation

Using the CAZymes Analysis Toolkit (CAT) (Park et al., 2010), differentially expressed genes related to carbohydrate active enzyme genes were annotated in the Carbohydrate Enzyme Database.^4^

### 2.8 Quantitative real-time PCR (qRT-PCR) validation of RNA-seq data

The expression of selected CAZymes genes were validated using qRT-PCR with three biological replicates and three technical replicates per biological replicate. Based on the results of GO and KEGG analysis, we selected five genes with known or predicted carbohydrate activity and responding to pH. qRT-PCR was carried out using a CFX96 Real-Time System (BIO-RAD) according to the manufacturer’s instructions, and SYBR green as the fluorescent dye (Libert et al., 2015). The primers used are listed in Supplementary Table S1. Internal Transcribed Spacer was used as the internal control gene. Genes were considered differentially expressed when fold change ≥1.5 or ≤ 0.667 and *p* ≤ 0.05.

### 2.9 Statistical analyses

Differences in PGP properties and qPCR expression levels between treatments were tested using analysis of variance (ANOVA) and Tukey *post hoc* test in SPSS 23.0. Differences were considered statistically significant at *p* < 0.05. Before the tests, the normality of distribution and homogeneity of variance were tested.

## 3 Results

### 3.1 The effect of pH on *Rhizopus delemar* growth and PGP capacity

**Figure 1:**
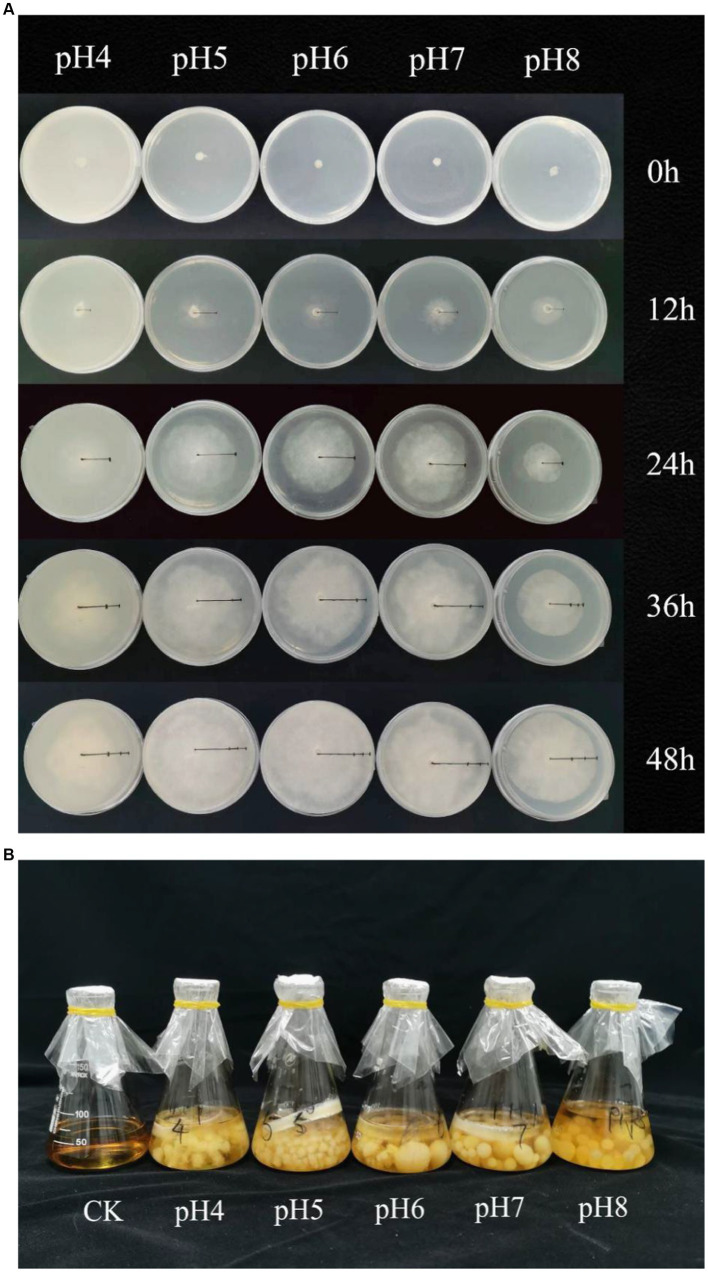
**(A)** The mycelium growth of *Rhizopus delemar* in PDA under different pH conditions (measured every 12 h). **(B)** The mycelium growth of *Rhizopus delemar* PDB under different pH conditions.

The mycelium biomass and growth rate were highest at pH 7, followed by pH 6 and pH 5 (*p* < 0.05) (Figure 1; Table 1). The mycelium biomass was lowest at pH 4 and the growth rate at pH 4 and pH 8 (*p* < 0.05). The IAA concentration ranged from 10.0 to 10.4 μg/mL, and the phosphate solubilization from 4.17 to 6.36 μg/mL; both were highest at pH 7. Based on the halo diameters, siderophore production was highest at pH 7, followed by pH 6, pH 8, pH 5, and pH 4 (*p* < 0.05) (Table 1).

**Table 1:** The mycelium biomass, growth rate, IAA production, CAS degradation, and phosphorus solubilization capacity of *Rhizopus delemar* grown at different pH levels.

	Mycelium biomass (g)	Mycelial growth rate (cm/h)	IAA production (μg/mL)	CAS degradation (cm/r)	Phosphorus solubilization capacity (μg/mL)
pH 4	7.79 ± 0.18e	0.050 ± 0.02d	10.0 ± 0.87e	0.90 ± 0.02e	5.35 ± 0.12d
pH 5	10.7 ± 0.22c	0.060 ± 0.01c	10.1 ± 0.76d	1.00 ± 0.07d	6.15 ± 0.14c
pH 6	11.5 ± 0.28b	0.045 ± 0.02b	10.3 ± 0.88b	1.30 ± 0.05b	6.23 ± 0.17b
pH 7	12.8 ± 0.27a	0.057 ± 0.01a	10.4 ± 1.04a	1.50 ± 0.08a	6.36 ± 0.18a
pH 8	9.54 ± 0.25d	0.025 ± 0.03d	10.2 ± 0.75c	1.10 ± 0.08c	4.17 ± 0.12e

Data are mean value ± SE (*n* = 3). Different letters in a column denote statistically significant differences (*P* < 0.05). CAS, Chrome azurol S; IAA, indole-3-acetic acid.

**Figure 2:**
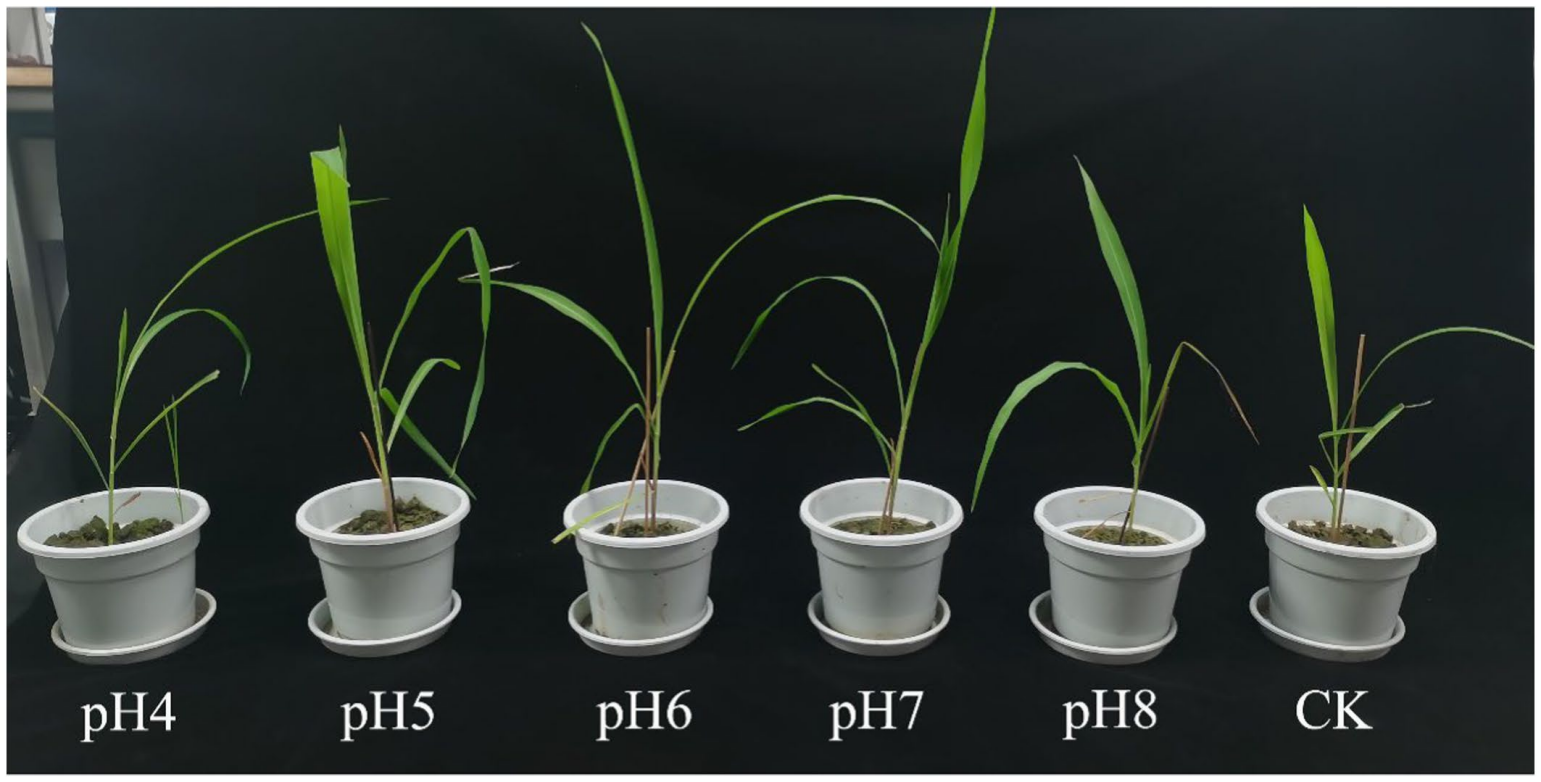
Potted plant of *Rhizopus delemar* corn under different pH conditions.

In the pot experiment, plant height and shoot weight were highest at pH 7 and lowest at pH 4 and pH 5 (*p* < 0.05) (Figure 2; Table 2). Intriguingly, roots were longest at pH 8, followed by pH 7 and 5, pH 6, and pH 4, whereas the number of roots was greatest at pH 5 to pH 7, followed by pH 8, and pH 4 (*p* < 0.05). Leaf area, fresh and dry root weight and dry shoot weight were biggest at pH 7 and smallest at pH 4 (*p* < 0.05).

**Table 2:** The properties of corn seedlings treated with the fermentation broth of *Rhizopus delemar* grown at different pH conditions.

	Plant height (cm)	Root length (cm)	Leaf area (cm^2^)	Root number	Aboveground fresh weight (g)	Fresh root weight (g)	Aboveground dry weight (g)	Dry root weight (g)
CK	44.8 ± 1.39d	15.5 ± 2.43e	48.4 ± 9.31d	9.67 ± 1.53c	2.06 ± 0.59c	0.229 ± 0.066d	0.225 ± 0.054d	0.100 ± 0.024d
pH 4	46.6 ± 2.42c	17.3 ± 1.03d	46.8 ± 3.42d	7.67 ± 0.580d	1.99 ± 0.015c	0.221 ± 0.029d	0.201 ± 0.014e	0.089 ± 0.020e
pH 5	47.0 ± 3.39c	23.1 ± 1.13b	50.8 ± 2.54d	12.7 ± 1.08a	2.26 ± 0.016c	0.251 ± 0.020c	0.234 ± 0.011c	0.104 ± 0.045d
pH 6	52.7 ± 3.31b	18.5 ± 1.09c	72.3 ± 3.31b	12.7 ± 1.04a	3.00 ± 0.017b	0.333 ± 0.019b	0.276 ± 0.012b	0.123 ± 0.045b
pH 7	56.0 ± 2.79a	23.5 ± 1.76b	85.5 ± 3.30a	12.7 ± 1.08a	3.74 ± 0.017a	0.415 ± 0.026a	0.318 ± 0.014a	0.141 ± 0.015a
pH 8	51.2 ± 3.35b	24.6 ± 1.30a	68.8 ± 3.21c	10.0 ± 1.65b	2.71 ± 0.015b	0.301 ± 0.024b	0.249 ± 0.013c	0.111 ± 0.033c

Data are mean value ± SE (*n* = 3). Different letters in a column denote statistically significant differences (*P* < 0.05).

### 3.2 *Rhizopus delemar* gene expression under different pH treatments

**Figure 3:**
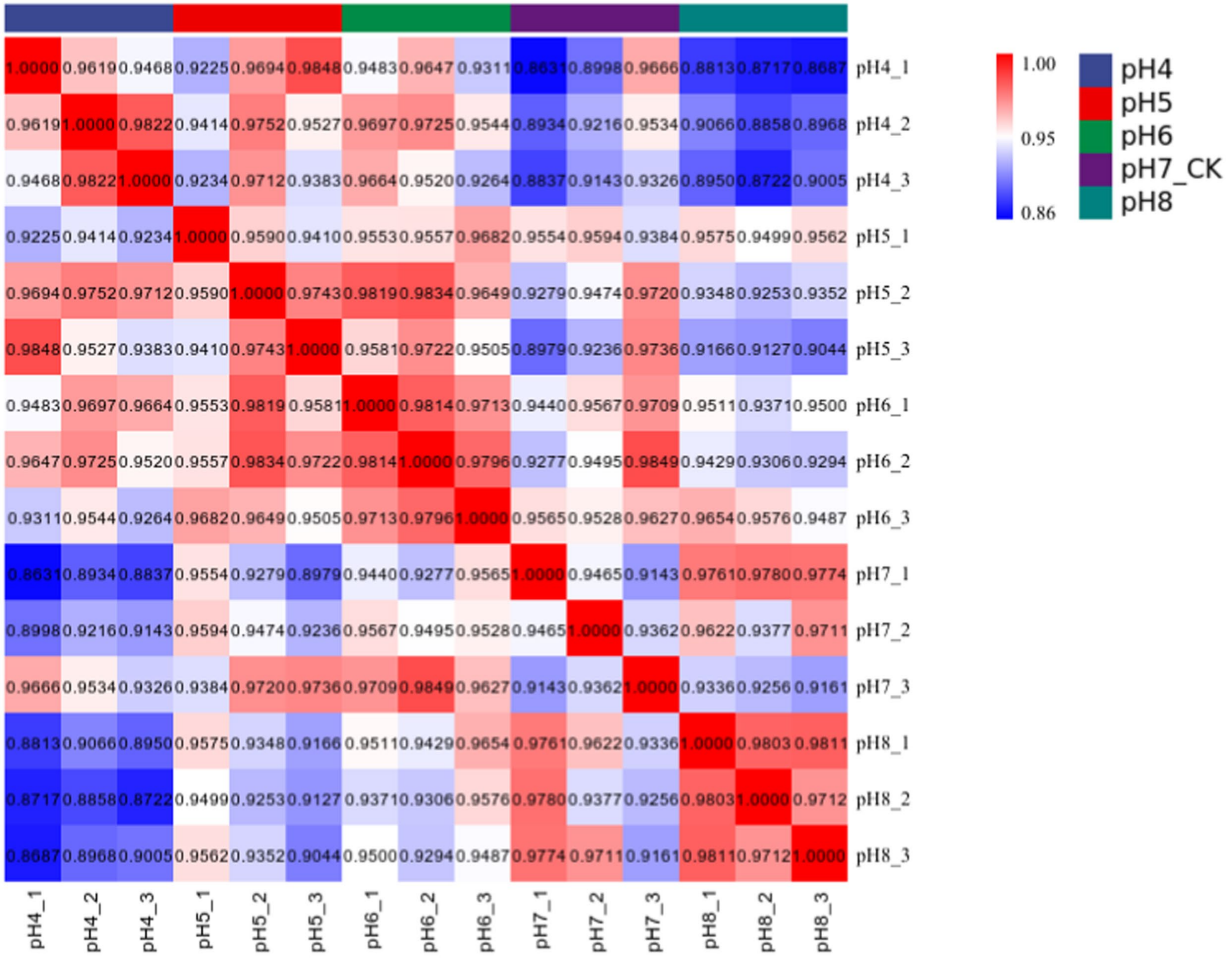
Pearson correlation coefficient was used to represent the correlation of gene expression levels between samples.

**Figure 4:**
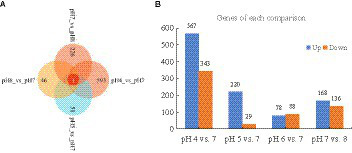
**(A)** Unique DEGs in the comparisons between pH 7 and the other pH values; **(B)** The number of up-regulated and down-regulated DEGs in the comparisons.

**Figure 5:**
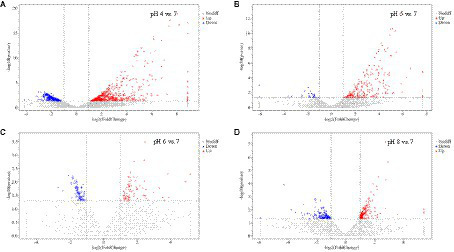
Volcanic expression distribution of DEGs in *Rhizopus delemar* transcriptome at different pH levels. **(A)** pH 4 vs. 7, **(B)** pH 5 vs. 7, **(C)** pH 6 vs. 7, **(D)** pH 8 vs. 7).

Approximately 94% of the reads passed the quality filtering, and approximately 96% of the filtered reads were mapped to the *R. delemar* genome. We obtained about 4.2, 4.0, 4.3, and 4.4 million reads for the different pH treatment *R. delemar*, and 4.7 million reads for the pH 7 (Supplementary Table S2). Within the replicates, the correlation in gene expression varied from 0.86 to 0.98 (Figure 3). In total, 1,629 DEGs were detected between the pH 7 and the other pH values (|log2FoldChange|>1) (Figures 4,5; Supplementary Table S1). In the comparisons, the number of unique DEGs ranged from 593 in the pH 4 vs. pH 7 to 46 in the pH 6 vs. pH 7; most of the DEGs at pH4 and pH 5 were up-regulated genes compared to pH 7 (Figure 4).

At pH 4 vs. pH 7, 567 and 343 genes were significantly upregulated and downregulated, respectively. Upregulated genes included serine/threonine-protein kinase PAK 1 (G6F53_003097), 5′-nucleotidase doman-containing protein (G6F53_003201), transforming growth factor-beta-induced protein (G6F53_007702), ABC transporter G family member (G6F53_007454), peptidoglycan-N-acetylglucosamine deacetylase (G6F53_000518), alpha-mannosidase (G6F53_007010), chitin synthase (G6F53_007011); downregulated genes included 12-oxophytodienoate reductase (G6F53_002340), and cAMP-dependent protein kinase (G6F53_000024), nucleolar MIF4G domain-containing protein (G6F53_004769). At pH 5 vs. pH 7, 220 and 29 genes were significantly upregulated and downregulated, respectively. Upregulated genes included hypothetical proteins (G6F53_002001), mesaconyl-C (4)-CoA hydratase (G6F53_000105), and 5′-nucleotidase domain-containing protein (G6F53_003201); downregulated genes included transposable element Tc1 transposase (G6F53_013486) and hypothetical protein (G6F53_000790). At pH 6 vs. pH 7, 78 and 88 genes were significantly upregulated and downregulated, respectively. Upregulated genes included tigger transposable element-derived protein (G6F53_006194, G6F53_002255) and ammonium transporter (G6F53_000650); downregulated genes included proton-coupled amino acid transporter (G6F53_002419) and chitinase (G6F53_001950). At pH 8 vs. pH 7, 168 and 136 genes were significantly upregulated and downregulated, respectively. Upregulated genes included transposable element Tc1 transposase (G6F53_013294) and hypothetical proteins; downregulated genes included trimethylguanosine synthase (G6F53_011806) and iron transport multicopper oxidase (G6F53_000791).

#### 3.2.1 Go enrichment analysis of DEGs of *Rhizopus delemar*

**Figure 6:**
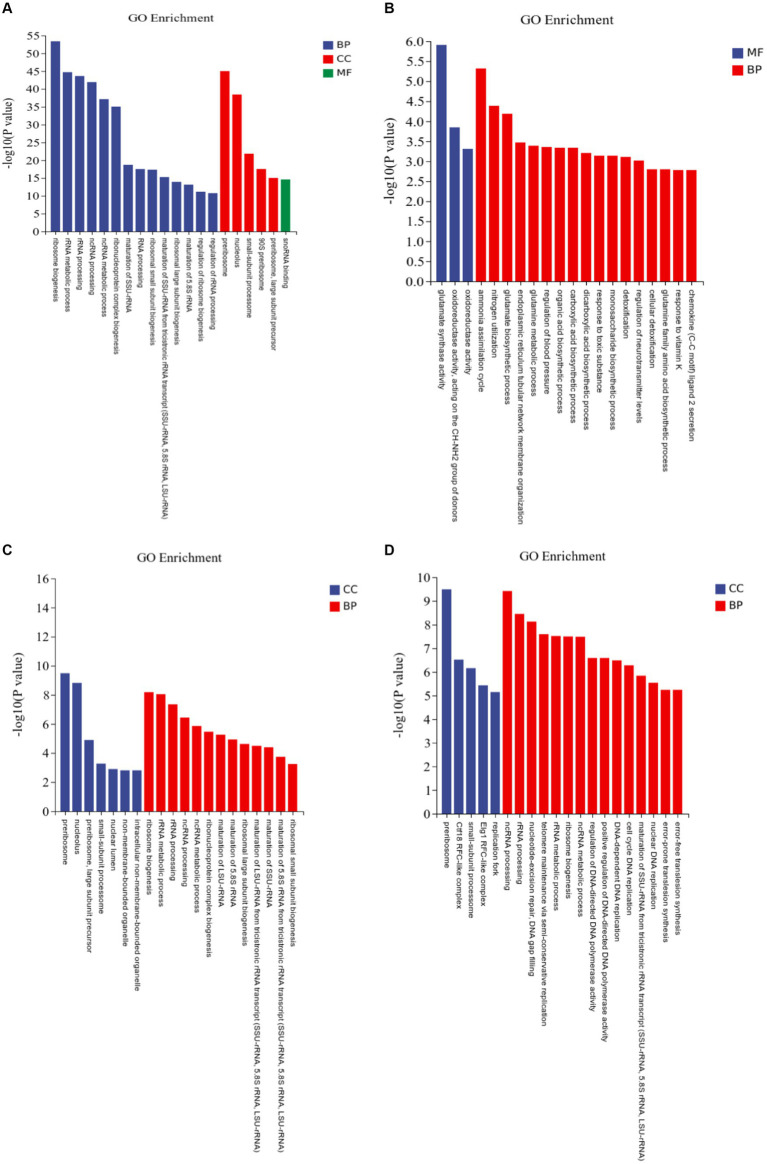
Gene Ontology (GO) assignments of differentially expressed genes (DEGs) in *Rhizopus delemar* transcriptome at different pH levels. **(A)** pH 4 vs. 7, **(B)** pH 5 vs. 7, **(C)** pH 6 vs. 7, **(D)** pH 8 vs. 7.

At pH 4 vs. pH 7, DEGs were classified into 839 molecular function (MF), 602 cell component (CC), and 5,426 biological process (BP) terms (Figure 6A; Supplementary Table S2). At pH 5 vs. 7, the DEGs were classified into 386 MFs, 298 CCs, and 2,676 BPs (Figure 6B). Ammonia assimilation cycle, nitrogen utilization, and glutamate biosynthetic process were enriched. At pH 6 vs. 7, the DEGs were classified into 376 MFs, 267 CCs, and 2,456 BPs (Figure 6C). Ribosome biogenesis, rRNA metabolic process, and rRNA processing were enriched. At pH 8 vs. 7, the DEGs were classified into 495 MFs, 397 CCs, and 2,972 BPs (Figure 6D).

Several up-regulated genes involved in plant growth promotion via auxin production were identified, including: auxin-responsive protein SAUR78, indole-3-acetic acid-amido synthetase and protein RALF-like 24 precursor, which were mainly included in GO terms: indole-containing compound metabolic process (GO: 0042430, GO: 0042436), positive regulation of cell growth (GO: 0030307) and positive regulation of cell development (GO: 0010720).

#### 3.2.2 KEGG pathway enrichment analysis of DEGs of *Rhizopus delemar*

**Figure 7:**
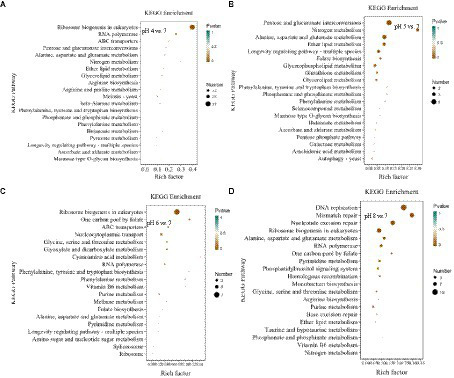
KEGG pathway enrichment analysis of differentially expressed genes (DEGs) in *Rhizopus delemar* transcriptome at different pH levels. **(A)** pH 4 vs. 7, **(B)** pH 5 vs. 7, **(C)** pH 6 vs. 7, **(D)** pH 8 vs. 7. KEGG, Kyoto Encyclopedia of Genes and Genomes.

In the KEGG pathway enrichment analysis, the DEGs at pH 4 vs. 7 were assigned to 286 pathways; at pH 5 vs. 7, to 55 pathways; at pH 6 vs. 7, to 34 pathways; and at pH 8 vs. 7, to 48 pathways (Figure 7; Supplementary Table S3).

At pH 4 vs. 7, the eukaryotic pathway ribosome biogenesis (ko03008) contained 56 up-regulated genes and 37 down-regulated genes. Glycolysis/gluconeogenesis (ko00010) contained 2 up-regulated genes and 5 down-regulated genes, and pyruvate metabolism pathway (ko00620) contained 2 up-regulated genes and 6 down-regulated genes. At pH 5 vs. 7, the metabolism of pentose and glucuronate (ko00040) contained 5 up-regulated genes, the butanoate metabolism (ko00650) treatment contained 1 up-regulated gene. Alanine, the alanine, aspartate and glutamate metabolism (ko00250) contain 2 up-regulated genes and 2 down-regulated genes. At pH 6 vs. 7, among the enriched genetic information processing pathways, the ribosome biogenesis in the eukaryotes pathway (ko03008) contained seven downregulated genes, and one carbon pool by the folate pathway (ko00670) contained two downregulated genes. At pH 8 vs. 7, butyric acid metabolism (ko00650) treatment contained 1 up-regulated gene, propionic acid metabolism pathway (ko00640) treatment contained 1 up-regulated gene, and fructose and mannose metabolism (ko00051) treatment contained 1 up-regulated gene, and alanine, aspartate and glutamate metabolism pathway (ko00250) contained 2 up-regulated genes and 3 down-regulated genes.

The results implied that *R. delemar* gene expression was significantly affected by pH. Most of the DEGs were enriched in butyric acid metabolism, pyruvate metabolism pathway, glycolysis/ gluconeogenesis, propionic acid metabolism pathway and other CAZymes (*p* < 0.05).

### 3.3 Differentially expressed CAZymes genes

**Figure 8:**
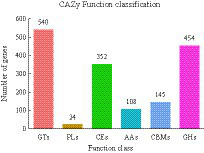
The classification of differentially expressed genes (DEGs) in *Rhizopus delemar* transcriptome at different pH levels into carbohydrate active enzyme (CAZymes) families.

A total of 1,623 genes of *R. delemar* transcriptome were identified as CAZymes genes (Supplementary Tables S4). Among these, 454 GHs, 108 AAs, 540 GTs, 352 CEs, 145 CBMs, and 24 PLs were identified (Figure 8). At pH 4 and 5 vs. 7 and at pH 7 vs. 8, 431 DEGs were related to CAZymes. At pH 6 vs. 7, 428 DEGs were related to CAZymes.

**Figure 9:**
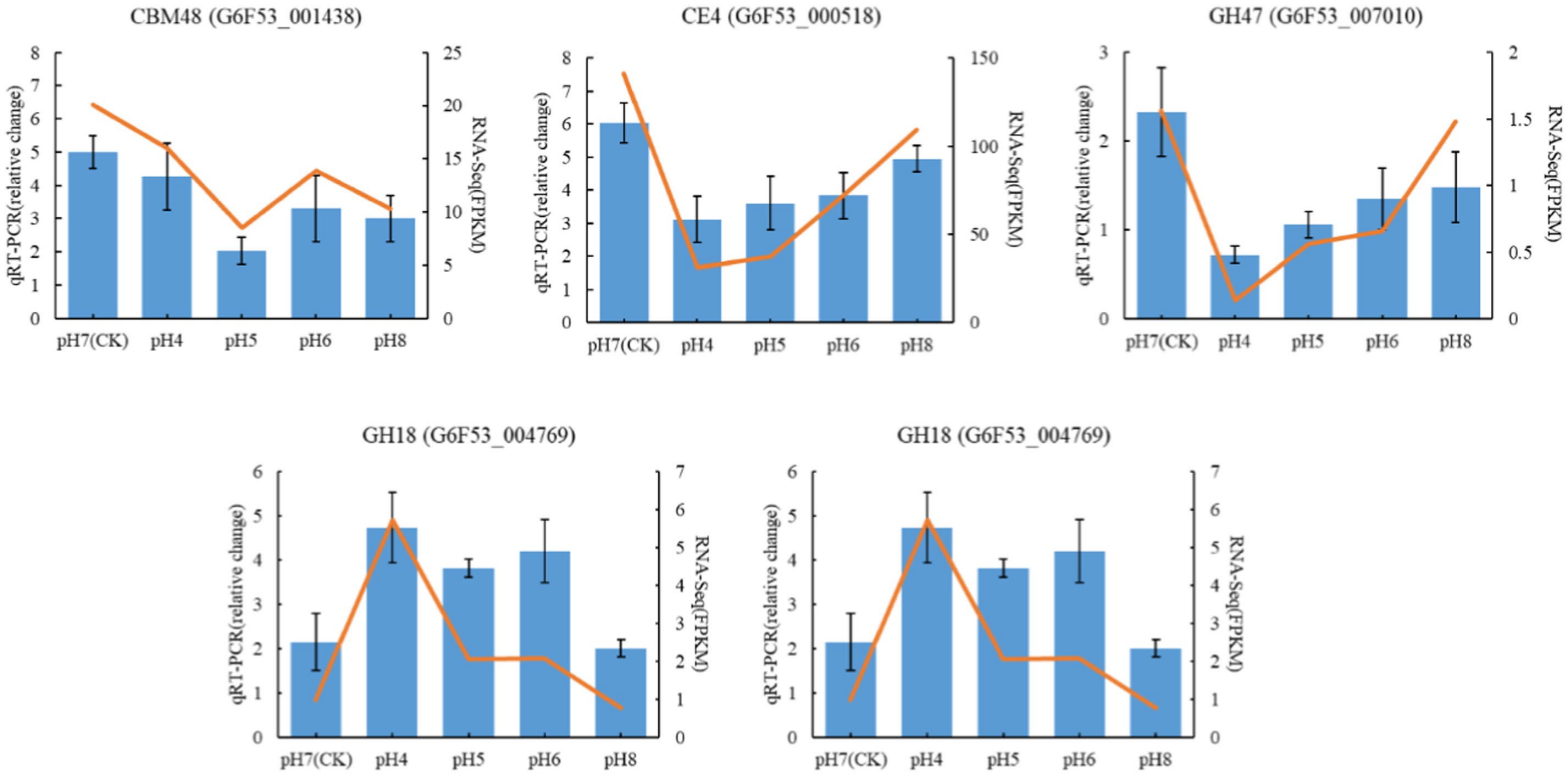
Validation of RNA-seq data by qRT-PCR Blue color bars represent the relative expression levels determined by qRT-PCR. Orange lines indicate the log2 fold change based on the read count values of the RNA-seq analysis. Error bars indicate standard errors of the means (*n* = 3).

The qRT-PCR gene expression levels of five CAZymes genes, chosen based on their biological roles, were in line with the differential expression detected in the transcriptomic analysis (Figure 9), supporting the validity of the results.

### 3.4 Enzyme assay

**Figure 10:**
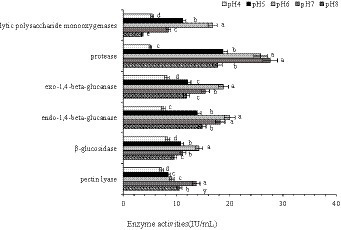
Lytic polysaccharide monooxygenase, protease, exo-1,4-beta-glucanase, endo-1,4-beta-glucanase, β-glucosidase, and pectin lyase activities in *Rhizopus delemar* grown at different pH levels. (*p* < 0.05).

To further reveal the effect of pH on CAZymes, endo-and exo-1,4-beta-glucanase, β-glucosidase, lytic polysaccharide monooxygenases, pectin lyase, and protease activities were determined. Endo-and exo-1,4-beta-glucanase, β-glucosidase are classified under glycoside hydrolase (GHs) group in Carbohydrate Enzyme database (Wierzbicka-Woś et al., 2019). In the transcriptomic analysis, the number of up-regulated GHs genes were greater than that of down-regulated genes. The enzyme activities of β-glucosidase and endo-and exo-1,4-beta-glucanase were lower at pH 4 and pH 8 compared with pH7, indicating that the activity of hydrolases were inhibited under acid–base conditions. We also found that the activity of pectin lyase, encoded by PLs family genes, was highest at pH 7 (Figure 10; Supplementary Table S4).

## 4 Discussion

Fungi can be used to produce valuable metabolites at industrial scale and provide great potential for biotechnology and agriculture, e.g., due to plant growth promoting (PGP) abilities. The PGP properties, and the related genes in zygomycetes have received little attention.

Generally, *R. delemar* grows best at a neutral pH, and the growth of *R. delemar* decreased below pH 3.5 and above pH 6.5 (Turgeman et al., 2016), consistent with the results of this study. pH can affect spore germination, transcriptional expression, and metabolism-related pathways of fungi (Han et al., 2011; Li et al., 2019). Likewise, pH is expected to affect the expression of PGP properties. Similar with plant growth promoting bacteria (Joshi et al., 2021), the *R. delemar* PGP characteristics, e.g., Phosphorus solubilization ability, were strongest at pH 7 (Liu et al., 2021). Even though the production of organic acids lowers the pH of the culture medium, an initial low pH possibly inhibits the PGP abilities.

In agreement with Xu et al. (2020), the plants grew poorly at pH 4 and pH 8. The effect on growth was possibly connected to CAZymes activities. The GHs families contain hydrolases such as endo-and exo-1,4-beta-glucanase (Mendonça et al., 2023), which play an important role in the ductility of cell wall and affect plant growth and development. We found that although the gene expression of GHs in CAZymes was up-regulated under pH stress, the endo-and exo-1,4-beta-glucanase and β-glucosidase activities were highest at pH 7. In addition, the better growth at pH 7 may be related to the *R. delemar*’s ability to dissolve more phosphorus at pH 7 than at pH 4 and pH 8. Compared with the root system, the aboveground part of the plant is more affected by soil phosphorus deficiency. Phosphorus deficiency directly inhibits plant growth by reducing enzyme activity, and indirectly inhibits plant growth by inhibiting leaf photosynthesis (Yang et al., 2022).

In line with the *in vitro* results, *R. delemar* grown at pH 7 had strongest PGP effect on seedlings. Although the concentration of IAA produced by *R. delemar* was not high, there were differences in the growth of plants. It may be that when plants are subjected to pH stress, the semi-permeability of the protoplast membrane disappears, and the cells are subjected to osmotic stress, which accelerated the process of the leaching of anions (Lager et al., 2010), resulting in a significant difference in plant height between pH 4 and pH 8. In our study, genes related to auxin biosynthesis and metabolism were found. These genes are responsible for the division, elongation and differentiation of plant cells and are closely related to plant growth (Matthes et al., 2019). Under pH stress, it may be the interaction between *Auxin/Índole-3-acetic acid* (Aux/IAA) and *Auxin response factor* (ARF). When the auxin concentration is low, the repressor protein Aux / IAA forms a complex with the ARF protein to regulate the expression of auxin-responsive genes and prevent them from acting as transcription factors (Ortiz et al., 2019), which is consistent with this study.

In this study, the identified DEGs revealed that pH influence *R. delemar* gene expression. KEGG pathway analysis showed that differentially expressed genes were mainly involved in ribosomal biogenesis of the eukaryotes pathway. The differentially expressed genes of ribosomal biogenesis pathway under pH stress were all down-regulated, indicating that *R. delemar* was constantly producing less new proteins in order to adapt to pH changes. The above pathways are basically carbohydrate metabolism processes. It provides carbon source and energy for the growth and reproduction of *R. delemar* through carbohydrate metabolism.

In the GO enrichment analysis, the number of differentially expressed genes in BP and CC were the largest, the number of down-regulated genes being higher than that of up-regulated genes, and the differentially expressed genes in ribosome biogenesis were significantly expressed. Ribosome biogenesis is regulated at several levels, including transcription of ribosomal genes and phosphorylation, methylation, and acetylation of constituent nucleolar factors, as well as the transport and interaction of these factors (Leary and Huang, 2001). In the comparison of pH 5 vs. pH 7, the GO enrichment of glutamate synthase metabolism was the most significant, and the glutamate differential gene was annotated as an acid ribosomal protein synthesis pathway, which indicated that the synthesis of acid protein would be promoted due to pH stress. Enrichment in pathways related to carbohydrate metabolism, transportation, catabolism, amino acid metabolism, and other nutrient-related processes indicates substantial environmental pressure due to pH changes for *R. delemar*.

The pH value has an important effect on pentose, glucuronic acid conversion, starch, and sucrose metabolism. Notably, CAZymes cellulase and hemicellulase play pivotal roles in cell wall polysaccharide hydrolysis, crucial for substrate degradation and microbial growth (Kala et al., 2017). CAZymes in carbohydrate metabolism are important for microbial growth, development, and metabolism, and the accumulation of microbial nutrients can be assessed by studying their activities (Wang et al., 2019). Glycoside hydrolases (GHs) include many enzymes that can hydrolyze glycosidic bonds in carbohydrates or between carbohydrate and non-carbohydrate molecules (Gupta et al., 2022). Among the CAZymes genes differentially expressed at pH 4 vs. 7, 78 and 35 genes encoding GHs were upregulated and downregulated, respectively, indicating their crucial role in providing adequate nutrition for *R. delemar*. Similarly, Fuglsang et al. (2000) reported that the GH18 gene may be related to chitinase activity; GH18 and CBM24 have been reported to bind to α-1,3-mutan, a mixed linked glucan from *Streptococcus* sp. Most differentially expressed, carbohydrate metabolism associated genes were downregulated at pH 4 vs. 8, indicating that carbohydrate metabolism in *R. delemar* was affected under acidic and alkaline conditions. Moreover, variations in the pH response within the GH18 enzyme subfamily were observed, showcasing a nuanced pH-dependent regulation within the enzyme subtypes.

In GHs that degrade cellulose, hemicellulose, chitosan, or arabinogalactan, the catalytic module is typically connected with one or more non-catalytic CBM that act independently (Veneault-Fourrey et al., 2014). Further research is needed to investigate whether the presence and location of CBM24 components affect the expression of the GH18 gene. In this study, one CBM12, two CBM13, three CBM30, and two CBM50 genes were upregulated, while one CBM1 and one CBM13 gene were downregulated at pH 4 vs. 7. CBM13 exhibits various sugar-binding specificities and is present in many CAZymes (Fujimoto, 2013). Most cellulose-binding domains attached to cellulolytic enzymes belong to CBM1 (Chen et al., 2014). On the cellulose surface, CBM1 enhances the hydrolysis of cellulose by increasing the concentration of effective enzymes (Takeda et al., 2015). The decrease in CBM1 expression may suggest that lower pH levels require fewer excessive cellulose-degrading enzymes.

At pH 4 vs. 7, 4 CE1, 22 CE4, 3 CE8, 1 CE9, 5 CE10, and 3 CE16 genes were upregulated, and 9 CE1 and 16 CE10 genes were downregulated. CE4 family is the largest in the CE family, and the structure of CE4 enzymes from many bacterial species has been determined (Aragunde et al., 2018). CE16 acts on glucuronoxylan and is induced by endo-β-1,4-glucanase generated fragments (Biely et al., 2016). CE1 and CE10 family members have carboxylesterase and endo-1, 4-β-xylanase activity (Zhao et al., 2014) and exhibit substantial diversity in substrate specificity. CE10 enzymes can act on non-carbohydrate substrates. The CE10 α-helix facilitates binding with specific substrates, such as glutamic acid or aspartic acid. After the formation of active sites, changes in enzyme structure allow for varied functions (Grams and Ospina-Giraldo, 2019), potentially explaining the mix of up- and downregulated genes within the CE10 family.

GTs of catalyze glycosidic bond formation to produce glycosides, participating in oligosaccharide, polysaccharide, and glycoconjugate biosynthesis. These enzymes transfer glycosyl groups using activated donor glycophosphate to form glycosidic bonds with specific receptor molecules (Williams et al., 2008). In our study, GT2, GT15, and GT20 were upregulated at pH 6, and GT1, GT2, GT8, and GT71 were downregulated. Lytic polysaccharide monooxygenases (PLs) use a β-elimination mechanism to split polysaccharides containing alduronic acid, generating unsaturated polysaccharides (El Kaoutari et al., 2013). In this study, pH stress potentially induced significant enzyme synthesis. Two or more polysaccharide utilization groups belonging to the PL7 family possibly promoted substrate degradation and synthesis. However, the specific functions of PLs remain to be clarified.

AAs of CAZymes are currently categorized into eight ligninolytic enzyme families and three lytic polysaccharide monooxygenase families, mainly based on amino acid sequence similarities. AAs include enzymes that cleave glycosidic bonds through an oxidation mechanism (Levasseur et al., 2013). Seven AA families were detected in *R. delemar*, including AA1, AA2, AA3, AA5, AA6, and AA7.

Consistent with the findings of Ikasari and Mitchell (1996), the activities of endo-and exo-1,4-beta-glucanase, β-glucosidase, lytic polysaccharide monooxygenases, pectin lyase, and protease in *R. delemar* remained highest at pH 6 or 7.

In conclusion, pH affected the growth and PGP properties of *R. delemar*, with the optimum pH being 7. In addition, pH affected the expression and activity of enzymes related to carbohydrate metabolism, contributing to the nutritional requirements of *R. delemar*. However, further clarification of the functions of differentially expressed genes is warranted.

## Data availability statement

The datasets presented in this study can be found in online repositories. The names of the repository/repositories and accession number(s) can be found in the article/Supplementary material.

## Author contributions

JL: Data curation, Validation, Writing – original draft, Writing – review & editing. YC: Validation, Writing – review & editing. SL: Validation, Writing – review & editing. DL: Data curation, Writing – review & editing. HT: Data curation, Writing – review & editing. QX: Data curation, Writing – review & editing. KZ: Data curation, Writing – review & editing. XY: Data curation, Writing – review & editing. QC: Data curation, Writing – review & editing. HF: Data curation, Writing – review & editing. LZ: Data curation, Writing – review & editing. PP: Writing – original draft, Writing – review & editing. YG: Writing – original draft, Writing – review & editing.

## Footnotes

^1^http://kobas.cbi.pku.edu.cn/

^2^https://www.kegg.jp/kegg/

^3^http://www.geneontology.org/

^4^http://www.cazy.org
