# Supplementary material for: Transcriptomic analysis and carbohydrate metabolism-related enzyme expression across different pH values in Rhizopus delemar
Source: Front Microbiol. 2024 Mar 6;15:1359830. doi: 10.3389/fmicb.2024.1359830 (PMC10953822; doi:10.3389/fmicb.2024.1359830)
Supplement: Supplementary file 1 [file Table_1.DOCX]

**Supplementary materials**

**Transcriptomic Analysis and Carbohydrate Metabolism-related Enzyme Expression Across Different pH Values in *Rhizopus delemar***

Jinpeng Liang^1†^, Yulan Chen ^1,3†^, Qinyan Yang^1†^, Sisi Li^1^, Yong Wang^1,3^, Hong Tian^1^, Quanju Xiang^1^, Ke Zhao^1^, Xiumei Yu^1^, Qiang Chen^1^, Hongzhu Fan^2^, Lingzi Zhang^1^, Petri Penttinen^1^*, Yunfu Gu^1^*

^1^Department of Microbiology, College of Resources, Sichuan Agricultural University, Chengdu 611130, China

^2^Institute of Agricultural Resources and Environmental Science, Sichuan Academy of Agricultural Sciences, Chengdu 610066, China

^3^Liangshan Tobacco Corporation of Sichuan Province, Xichang, 615000, China

| Table S1. The sequences of seven CAZymes genes primer. | |
| --- | --- |
| **Target genes** | **Primer sequences** |
| G6F53_000518 | Forward: 5'AAATGATTTTTTTTACTATATGAATAAT3' |
|  | Reverse: 5'CTTCCTTTTTTTCTTTTTC3' |
| G6F53_007011 | Forward: 5'ATGTGATATAGATTCATGAAAAGAAGAAT3' |
|  | Reverse: 5'GCAATATAATCCCTCCCTCC3' |
| G6F53_007010 | Forward: 5'TTTATTAAAGAGGGGGAGTGG3' |
|  | Reverse: 5'CCACTAGTTACAACATCAATATCATAGT3' |
| G6F53_004769 | Forward: 5'AAGGTGAGGAGAAATGAGG3' |
|  | Reverse: 5'AGCTCCTTCCATATGTTAAATA3' |
| G6F53_001438 | Forward: 5'AAATTCTAAGGACGTTTAGTGGAGATATG3' |
|  | Reverse: 5'TATCTTCCCCGACTTCTGG3' |

Table S2. Numbers of reads in the transcriptomes of *R. delema* under different pH conditions (pH4, pH5, pH6, pH7, pH8).

| Sample | Raw Reads | Clean Reads | Clean Reads (%) | Total Mapped Reads | Mapped Ratio | Mapped to Gene | Mapped to Exons |
| --- | --- | --- | --- | --- | --- | --- | --- |
| pH4 | 42,283,168 | 39683063 | 93.8 | 38242283 | 96.4 | 95.1 | 99.8 |
| pH5 | 40,070,320 | 37524162 | 93.7 | 36223747 | 96.5 | 94.9 | 99.8 |
| pH6 | 43,530,779 | 40812398 | 93.8 | 39560597 | 96.9% | 94.9 | 99.7 |
| pH7 | 47,077,541 | 44241764 | 94.0 | 42739348 | 96.7 | 94.3 | 99.7 |
| pH8 | 44,804,012 | 42078846 | 93.9 | 40853593 | 97.1 | 94.3 | 99.7 |
